# The Essential Oil from Acori Tatarinowii Rhizome (the Dried Rhizome of *Acorus tatarinowii* Schott) Prevents Hydrogen Peroxide-Induced Cell Injury in PC12 Cells: A Signaling Triggered by CREB/PGC-1*α* Activation

**DOI:** 10.1155/2020/4845028

**Published:** 2020-03-09

**Authors:** Lu Yan, Gail Mahady, Yiyun Qian, Pingping Song, Tunyu Jian, Xiaoqin Ding, Fuqin Guan, Yu Shan, Min Wei

**Affiliations:** ^1^Institute of Botany, Jiangsu Province and Chinese Academy of Sciences, Nanjing 210014, China; ^2^Jiangsu Key Laboratory for Research and Utilization of Plant Resources, Nanjing 210014, China; ^3^The Jiangsu Provincial Platform for Conservation and Utilization of Agricultural Germplasm, Nanjing 210014, China; ^4^Department of Pharmacy Practice, College of Pharmacy, University of Illinois at Chicago, Chicago, IL 60612, USA

## Abstract

Acori Tatarinowii Rhizome (ATR, the dried rhizome of *Acorus tatarinowii* Schott), a well-recognized traditional Chinese herbal medicine, is prescribed to treat neurological disorders. The essential oil is considered as the active fraction of ATR, and the neuroprotection of ATR essential oil (ATEO) is proven, including the protection against oxidative stress. However, the cellular mechanism of ATEO against oxidative stress has not been fully illustrated. In this study, to investigate the cellular mechanism of ATEO, the cytoprotective effect of ATEO against H_2_O_2_-induced injury was revealed in PC12 cells. ATEO treatment increased the viability of cells affected by H_2_O_2_-mediated injury, inhibited reactive oxygen species (ROS) accumulation, and induced the expression of several antioxidant proteins (SODs, GPx, and UCPs). The cytoprotective effect of ATEO was related to upregulation of peroxisome proliferator-activated receptor gamma coactivator 1-alpha (PGC-1*α*) expression, which was counteracted by PGC-1*α* specific knockdown. Using inhibitor of protein kinase A (PKA), we found that cAMP-response element binding protein (CREB) activation was involved in ATEO-induced PGC-1*α* expression. Taken together, we suggest that ATEO effectively prevents H_2_O_2_-induced cell injury possibly through the activation of CREB/PGC-1*α* signaling in PC12 cells. The results provide a molecular insight into the effect of ATEO on cytoprotection against oxidative stress.

## 1. Introduction

Oxidative stress is implicated in the pathogenesis of several neurological disorders, including Alzheimer disease, Parkinson disease, amyotrophic lateral sclerosis, stroke, and depression [1–3]. Excessive reactive oxygen species (ROS) production appears to contribute to cellular damage, impairment of the DNA repair system, and mitochondrial dysfunction, all of which are known as key factors in these neurological disorders [4–6]. The first line of defense against ROS in cells is the detoxifying enzymes that scavenge ROS, including superoxide dismutases (SODs), glutathione peroxidase (GPx), and catalase [7–9]. Another line of defense is the uncoupling proteins (UCPs) that limit mitochondrial ROS overproduction [10]. These proteins have a significant impact on ROS metabolism and the modulation of the antioxidant defense system prevents ROS-mediated damage in neuronal cells.

Peroxisome proliferator-activated receptor gamma coactivator 1-alpha (PGC-1*α*) is an inducible transcription coactivator that regulates the genes involved in energy metabolism [11, 12]. In addition to modulating energy metabolism, there have been many reports of the beneficial effects of PGC-1*α* as a master regulatory protein of antioxidant capacity [13, 14]. Studies of neurological disorders reported that the loss of PGC-1*α* expression was closely related to ROS accumulation and neuron loss, while upregulation of PGC-1*α* expression induced the expression of SODs, GPx1, and UCPs and therefore contributed to ROS metabolism. Moreover, the cAMP-response element binding protein (CREB) was shown to function as an upstream activator of PGC-1*α* gene expression to promote neuronal survival [15, 16]. On the basis of these studies, the activation of CREB/PGC-1*α* signaling suggests a novel and effective neuroprotective target involving oxidative stress.

Acori Tatarinowii Rhizoma (ATR, the dried rhizome of *Acorus tatarinowii* Schott) has been one of the most important traditional herbal medicines in China for thousands of years [17, 18]. The application of ATR is used for the treatment of neurological disorders, acting on regaining consciousness, tranquilizing the mind, eliminating dampness, and invigorating the circulation of blood. It is reported that the extracts of ATR enhance neurogenesis and neuroprotection in both animal and clinical studies [19–21]. According to Chinese Pharmacopoeia, the major active fraction of ATR is the essential oil [22]. Recent studies have reported the application of ATR essential oil (ATEO) in neuroprotection, including the protection against oxidative stress [23, 24]. However, the cellular mechanism of ATEO against oxidative stress in neuronal cells has not been fully elucidated.

In this study, the cellular mechanism of ATEO against oxidative stress was investigated. PC12 cells were selected as the cell model due to their phenotypic characteristics of sympathetic neurons [25, 26]. We evaluated whether ATEO had a beneficial effect on H_2_O_2_-stressed PC12 cells, including a PGC-1*α*-mediated increase of cell viability, decrease of ROS level, and upregulation of antioxidant protein expression. We hypothesized that the activation of CREB, which was stimulated by ATEO, was one of the molecular mechanisms for the induction of PGC-1*α* gene expression. Therefore, we investigated whether ATEO-dependent activation of CREB/PGC-1*α* played a crucial role in the protective effect against H_2_O_2_-induced injury in PC12 cells.

## 2. Materials and Methods

### 2.1. Reagents and Chemicals

*α*-Asarone (>98%) and *β*-asarone (>98%) were purchased from Weikeqi-biotech (Chengdu, China). Ultrapure water was prepared from a Milli-Q purification system (Millipore, Molsheim, France). 3-(4,5-Dimethyl-2-thiazolyl)-2,5-diphenyl-2H-tetrazolium bromide (MTT) (>98%), H_2_O_2_, and other reagents were from Sigma-Aldrich (St. Louis, MO).

### 2.2. Preparation of ATEO

The plant materials were obtained from Bozhou Market, Anhui, China, and were morphologically authenticated by Dr. Min Wei, Institute of Botany, Jiangsu Province and Chinese Academy of Sciences, Nanjing. The corresponding voucher specimens were deposited in Research Center of Medicinal Plants of Institute of Botany, Jiangsu Province and Chinese Academy of Science. The plant materials were tested for quality according to the requirements of Chinese Pharmacopeia (2015 Edition). In preparing the essential oil, 100 g of the plant materials was minced and soaked in 800 mL of water for 1 h. The mixture was submitted to hydrodistillation in a volatile oil extractor for 8 h [22]. The resultant ATEO was collected, dried over anhydrous sodium sulfate, and stored at −20°C.

For relative quantification of ATEO, GC/MS analyses were performed on an Agilent 7890A GC system equipped with a 5975C EI-MS system (Agilent Technologies, Santa Clara, CA). The chromatographic separation was conducted on an Agilent HP-5MS column (30 m × 0.25 mm, 0.25 *μ*m) using the following temperature profile: 60°C for 0 min, 60°C–120°C at 5°C/min, held for 1 min, 120°C–150°C at 1°C/min, held for 3 min, and 150°C–280°C at 20°C/min, held for 2 min. The injection volume was 2 *μ*L. The ionization was performed in the electron impact mode at 70 eV. The ion source temperature was 230°C and the interface temperature was 250°C. Mass spectra were recorded in total ion chromatogram (TIC) mode (m/z 30–300) [27, 28]. The obtained chromatogram was automatically retrieved by NIST MS Search 2011, and the similarity was considered to be correct when the similarity was greater than 90%. For absolute quantification of ATEO, HPLC analyses were performed on an Agilent 1200 series HPLC system (Agilent Technologies). The samples were analyzed on an Agilent DAD detector G1315C with a wavelength of 270 nm. A Dikma Diamonsil Plus C18 column (4.6 × 250 mm, 5 *μ*m, Dikma Technologies, Beijing, China) maintained at ambient room temperature was selected. The mobile phase condition was recorded as follows: water (A) and acetonitrile (B). The flow rate was 1.0 mL/min with injection volume of 5 *μ*L. 0–10 min, linear gradient 70.0–55.0% (A); 10–30 min, isocratic gradient 55.0% (A); 30–40 min, linear gradient 55.0–25.0% (A); and 40–45 min, isocratic gradient 25.0% (A). A preequilibration period of 6 min was used in each sample. *α*-Asarone and *β*-asarone were identified by comparison of their retention time (Rt) with those of known standards from the chromatograms. All values are in mean ± SD, *n* = 3. The established chemical parameters served as the control for repeatability of biochemical analyses.

### 2.3. Cell Culture

PC12 cells (ATCC® CRL-1721) were obtained from American Type Culture Collection (ATCC, Manassas, VA) and were cultured in Dulbecco's modified Eagle's medium (DMEM), supplemented with 6% fetal bovine serum (FBS) and horse serum (HS), 100 units/mL penicillin, and 100 *μ*g/mL streptomycin in a humidified CO_2_ (5%) incubator at 37°C [29]. Culture medium was changed every other day. All culture reagents were purchased from Thermo Fisher Scientific (Waltham, MA).

### 2.4. Drug Treatment

In mRNA and protein expression assays, PC12 cells were seeded in 6-well plates at a density of 1.5 × 10^5^ cells per well and stayed overnight. The cells were treated with different concentrations of ATEO (1.5, 5, and 15 *μ*g/mL) for 48 h. The culture medium was replaced 3 h prior to drug treatment with or without H89 (Sigma, 5 *μ*M). In cytoprotection study, the cells were pretreated with ATEO (1.5, 5, and 15 *μ*g/mL) for 48 h before exposure to H_2_O_2_ (400 *μ*M) for 1 or 24 h. ZLN005 (MCE, Monmouth Junction, NJ, 2 *μ*M) was used as a positive control. In phosphorylation study, PC12 cells were seeded in 12-well plates at a density of 2 × 10^5^ cells per well and stayed overnight. The cells were treated with ATEO (15 *μ*g/mL) at different time points (from 0, 5, 10, and 30 min) after 3 h starvation. The degree of phosphorylation was determined by their specific anti-phospho-kinase and total kinase antibodies. Results were presented as intensities of phospho-bands relative to total bands and expressed as folds.

### 2.5. siRNA Knockdown

RNA interference of PGC-1*α* was performed using a PGC-1*α* specific siRNA (S: 5′-CCG AGA AUU CAU GGA GCA ATT-3′, AS: 5′-UUG CUC CAU GAA UUC UCG GTT-3′, Sangon Biotech, Shanghai, China, RS2988). Briefly, PC12 cells were seeded in 6-well plates at a density of 1.2 × 10^5^ cells per well and stayed overnight. The cells were transfected with siRNA (50 nM) using Lipofectamine™ RNAiMAX transfection reagent (Thermo Fisher Scientific) according to the manufacturer's protocol. Cells transfected with NC-siRNA (S: 5′-UUC UCC GAA CGU GUC ACG UTT-3′, AS: 5′-ACG UGA CAC GUU CGG AGA ATT-3′, Sangon Biotech, RS2988) were used as controls for direct comparison.

### 2.6. Cell Viability Assay

Cell viability was assessed by MTT assay. PC12 cells were seeded in 96-well plates at a density of 6000 cells per well and stayed overnight. The cells were pretreated with ATEO (1.5, 5, and 15 *μ*g/mL) for 48 h before exposure to H_2_O_2_ (400 *μ*M) for 24 h [30]. ZLN005 (MCE, 2 *μ*M) was used as a positive control. Then the cells were incubated with MTT for another 3 h at 37°C, and the absorbance of 570 nm was measured in a microplate reader (Infinite 200 PRO plate reader, TECAN, Switzerland).

### 2.7. Hoechst Staining

The nuclear morphology was studied by using florescent nuclear dye H33258. PC12 cells were seeded in 6-well plates at a density of 1.2 × 10^5^ cells per well and stayed overnight. The cells were pretreated with ATEO (1.5 and 15 *μ*g/mL) for 48 h before exposure to H_2_O_2_ (400 *μ*M) for 1 h. Then the cells were stained with Hoechst 33258 (Sangon Biotech, 10 *μ*g/mL) for 10 min and fixed by 4% paraformaldehyde for another 10 min. The cells were observed under a fluorescence microscope (Olympus BX61, Japan). Images were magnified at ×200, bar = 20 *μ*m.

### 2.8. Measurement of ROS Amount

Level of intracellular ROS was assessed using 2,7′-dichlorodihydrofluorescein diacetate (DCFH-DA, Jiancheng Bioengineering, Nanjing, China). Labelling with probes was conducted on lived cells, but not fixed cells. PC12 cells were seeded in 96-well plates at a density of 6000 cells per well and stayed overnight. PC12 cells were pretreated with ATEO (1.5 and 15 *μ*g/mL) for 48 h before exposure to H_2_O_2_ (400 *μ*M) for 1 h. Then the cells were loaded with 10 *μ*M DCFH-DA for 1 h at 25°C and washed with PBS twice. The amount of intracellular ROS was detected by fluorometric measurement with excitation at 485 nm and emission at 530 nm (Infinite 200 PRO plate reader, TECAN).

### 2.9. Real-Time Quantitative PCR

Total RNA was isolated from cell cultures by RNA Isolation Kit (Vazyme, Nanjing, China) according to the manufacturer's instructions. The concentrations of RNAs were detected by UV absorbance at 260 nm. cDNA was reverse-transcribed from 1 *μ*g of samples of total RNA using RT SuperMix for qPCR (Vazyme), according to the protocol provided by the manufacturer. Real-time PCR was performed using SYBR Green Master Mix (Vazyme). The SYBR green signal was detected by qTOWER 2.0 (Analytic Jena AG, Germany). Primers used were the following: 18S-S: TGT GAT GCC CTT AGA TGT CC; 18S-AS: GAT AGT CAA GTT CGA CCG TC; PGC-1*α*-S: GGC TCA AGA GGG ACG AAT ACC G; PGC-1*α*-AS: CCA TAG CTG TCT CCA TCA TCC CG; UCP2-S: ACG ACC TCC CTT GCC ACT TCA C; UCP2-AS: CAA GCG GAG GAA GGA AGG CAT G; UCP3-S: TTG GAG CTG GTT TCT GTG C; UCP3-AS: CGT TCA TGT ATC GGG TCT T; GPx1-S: GGA CTA CAC CGA AAT GAA TGA TCT G; GPx1-AS: GAA GGT AAA GAG CGG GTG AGC; SOD1-S: CGG CTT CTG TCG TCT CCT TGC TT; SOD1-AS: AAC TGG TTC ACC GCT TGC CTT CT; SOD2-S: GCC AAG GGA GAT GTT ACA ACT CAG; SOD2-AS: GCA GTG GGT CCT GAT TAG AGC AG.

### 2.10. SDS-PAGE and Immunoblotting

After drug treatment, cells were solubilized in lysis buffer containing 0.125 M Tris-HCl, PH6.8, 4% SDS, 20% glycerol, and 2% 2-mercaptoethanol and analyzed immediately or stored as being frozen at −20°C. Proteins were separated on 8% SDS-polyacrylamide gels and transferred to a PVDF membrane (Millipore, Billerica, MA). The PVDF membrane was blocked with 5% fat-free milk in Tris-buffer saline/0.1% Tween 20 (TBS-T) and then incubated in the primary antibodies diluted in 2.5% fat-free milk in TBS-T over night at 4°C. The primary antibodies were as follows: anti-PGC-1*α* (Sangon Biotech, D162041, 1 : 1,000), anti-*β*-actin (Sangon Biotech, D110001, 1 : 5,000), anti-phospho-CREB (Cell Signaling, Danvers, MA, 9198, 1 : 1,000), and anti-CREB (Cell Signaling, 9197, 1 : 5,000). After that, the PVDF membrane was rinsed with TBS-T and incubated for 2 h at room temperature in peroxidase (HRP)conjugated anti-rabbit secondary antibody (Sangon Biotech, D110058, 1 : 5,000), diluted in 2.5% fat-free milk in TBS-T. After extensive washing with TBS-T, the immune complexes were visualized using the enhanced chemiluminescence (ECL) kit (Vazyme). The intensities of bands in control and samples, run on the same gel and under strictly standardized ECL conditions, were compared on an image analyzer, using a calibration plot constructed from a parallel gel with serial dilutions of one of the sample.

### 2.11. Statistical Analysis

All data were analyzed using one-way ANOVA or Student's *t*-test method. Differences with values of *p* < 0.05 were considered significant.

## 3. Results

### 3.1. Standardization of ATEO

**Figure 1:**
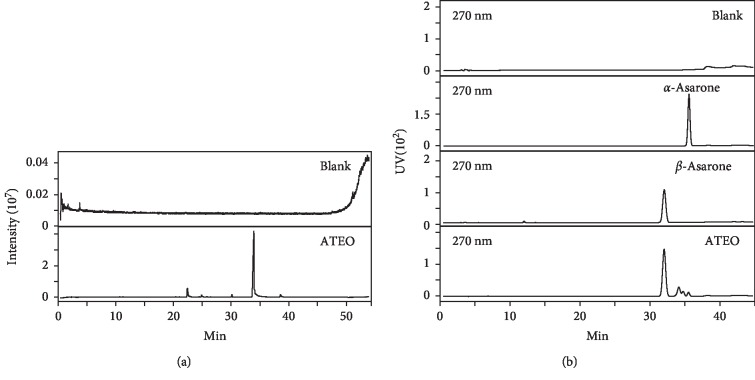
Total ion and HPLC chromatogram of ATEO. (a) The chromatographic method was described in Materials and Methods. The identification of 25 compounds was made by a MS detector. (b) The identification of *α*-asarone (270 nm) and *β*-asarone (270 nm) was made by a HPLC couple with a DAD detector. The detected wavelength was indicated. Representative chromatograms are shown, *n* = 3.

ATEO was prepared according to Chinese Pharmacopeia (2015 Edition), using 100 g of plant material and hydrodistillation in a volatile oil extractor for 8 h to extract the essential oil. The extraction efficiency was about 1.32 ± 0.28% (mean ± SD, *n* = 3). GC/MS was selected for relative quantification of ATEO. A total of 25 compounds were identified, representing 97.05% of the total oil (Figure 1(a) and Table 1). The main components were methyl eugenol (17.10%), *γ*-asarone (2.39%), *β*-asarone (68.25%), and *α*-asarone (4.65%). HPLC was selected for absolute quantification of ATEO (Figure 1(b)). The amounts in g/g of essential oil were 0.049 ± 0.002 for *α*-asarone and 0.687 ± 0.025 for *β*-asarone (mean ± SD, *n* = 3). The established chemical parameters served as control for repeatability of the below biochemical analyses.

### 3.2. ATEO Protected PC12 Cells from H_2_O_2_-Mediated Injury

**Figure 2:**
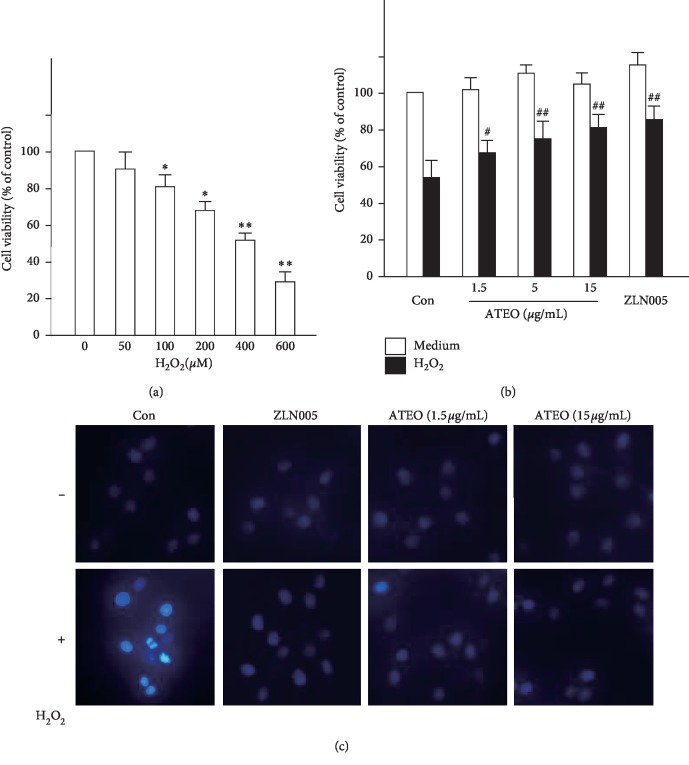
ATEO protected PC12 cells from H_2_O_2_-mediated injury. (a) PC12 cells were exposed to H_2_O_2_ at various concentrations (0–600 *μ*M) for 24 h for cytotoxicity test. (b) PC12 cells were pre-treated with ATEO (1.5, 5 and 15 *μ*g/mL) for 48 h before addition of H_2_O_2_ (400 *μ*M) for cytotoxicity test. (c) Nuclear morphological changes of H_2_O_2_-treated PC12 cells were shown. PC12 cells were pre-treated with ATEO at low and high concentrations (1.5, 15 *μ*g/mL) and ZLN005 (2 *μ*M) for 48 h before addition of H_2_O_2_ (400 *μ*M) for 1 h. Then the cultures were washed with PBS, stained with Hoechest 33258 and fixed with 4% paraformaldehyde. Bar = 20 *μ*m. Representative views were shown, *n* = 3. Cell viability was detected by MTT assay. Data are expressed as mean ± SEM, where *n* = 5. ^*∗*^*p* < 0.05, ^*∗∗*^*p* < 0.01 compared with the control group; ^#^*p* < 0.05, ^##^*p* < 0.01 compared with the H_2_O_2_-treated group.

To explore the role of ATEO in cytoprotection, the effects of ATEO on H_2_O_2_-induced cell toxicity in PC12 cells were determined. A cell viability assay was performed to determine a safe concentration range (0–15 *μ*g/mL) of ATEO (the concentrations at which all extracts did not induce cell proliferation or death; see Supplementary [Supplementary-material supplementary-material-1]). Then, the PC12 cells were treated with increasing concentrations of H_2_O_2_ (0–600 *μ*M). The cytotoxicity of PC12 cells induced by H_2_O_2_ was demonstrated by a dose-dependent decrease of cell viability (Figure 2(a)). Treatment of the PC12 cells with 400 *μ*M of H_2_O_2_ reduced cell survival by 50%. However, when the cells were pretreated with ATEO (1.5, 5, and 15 *μ*g/mL) and then H_2_O_2_, ATEO significantly prevented the decrease in cell viability (Figure 2(b)) and change in nuclear morphology (Figure 2(c)). It was determined that the pretreatment time of ATEO for cytoprotection was 48 h (Supplementary [Supplementary-material supplementary-material-1]). Similar results were observed in pretreatment of ZLN005 (2 *μ*M), a PGC-1*α* activator with known antioxidant capability [26]. Additionally, ATEO-treated cultures did not show abnormal nuclear morphology in PC12 cells (Figure 2(c)), which was consistent with the finding in safe concentration range. These results suggested that ATEO possessed significant protective effects against H_2_O_2_-mediated injury in PC12 cells.

### 3.3. ATEO Suppressed ROS Production and had an Antioxidant Effect

**Figure 3:**
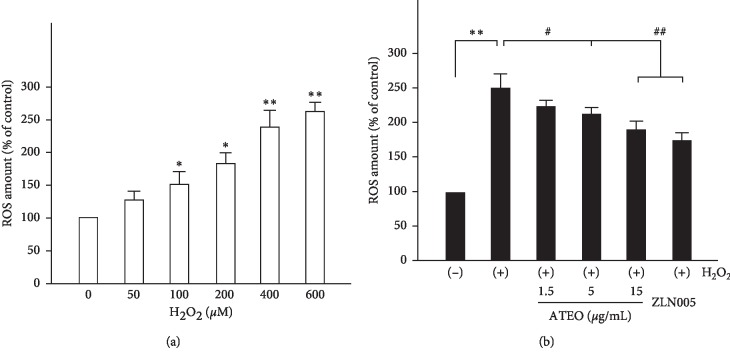
ATEO suppressed ROS production in H_2_O_2_-stressed PC12 cells. (a) PC12 cells were exposed to H_2_O_2_ (0–600 *μ*M) for 1 h The level of intracellular ROS was measured. (b) PC12 cells were pre-treated with ATEO (1.5, 5 and 15 *μ*g/mL) for 48 h and then exposed to H_2_O_2_ (400 *μ*M) for 1 h. ZLN005 (2 *μ*M) was used as a positive control. The results were in % of control (untreated culture). Data are expressed as mean ± SEM, *n* = 3. ^*∗*^*p* < 0.05, ^*∗∗*^*p* < 0.01 compared with the control group; ^#^*p* < 0.05, ^##^*p* < 0.01 compared with the H_2_O_2_-treated group.

Previous studies indicated that ATEO suppressed ROS production in astrocytes through the induction of ROS-detoxifying enzymes [24]. Here, we confirmed that intracellular ROS was regulated by ATEO in PC12 cells using a ROS probe, DCFH-DA. The level of intracellular ROS induced by H_2_O_2_ was demonstrated by a concentration-dependent increase (Figure 3(a)). At 400 *μ*M concentration of H_2_O_2_, the level of intracellular ROS was increased by ∼150%. Under such cytotoxic conditions, pretreatment of ATEO (1.5, 5, and 15 *μ*g/mL) significantly reduced intracellular ROS level (Figure 3(b)) in PC12 cells, where similar results were observed in pretreatment of ZLN005 (2 *μ*M).

### 3.4. ATEO Induced the Expression of Antioxidant Proteins

**Figure 4:**
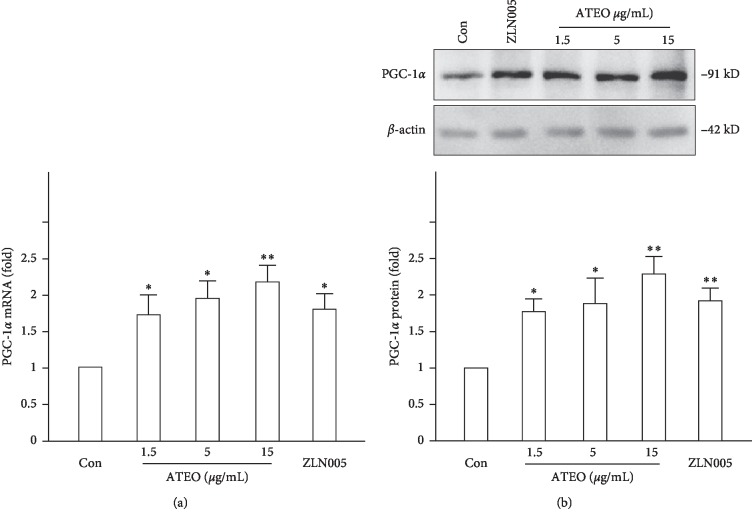
Treatment of PC12 cells with ATEO increased the expression of PGC-1*α*. PC12 cells were treated with ATEO (1.5, 5 and 15 *μ*g/mL) for 48 h. The mRNA and protein levels of PGC-1*α* were analyzed by real-time quantitative PCR (a) and Western blot assay (b), respectively. *β*-actin served as an internal control. ZLN005 (2 *μ*M) was used as a positive control. Data are expressed as fold of control (untreated culture), and mean ± SEM, where *n* = 3. ^*∗*^*p* < 0.05, ^*∗∗*^*p* < 0.01 compared with control group.

**Figure 5:**
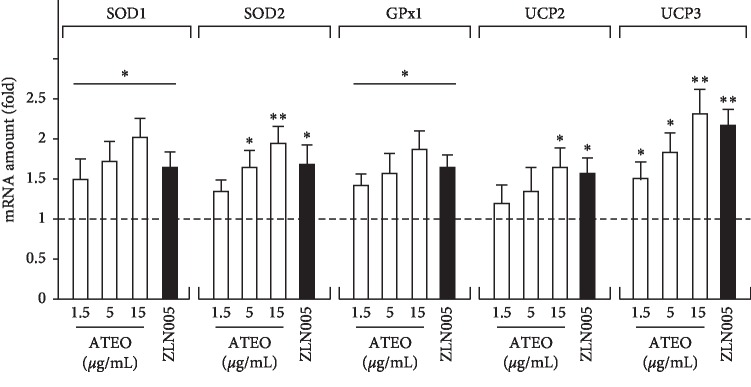
Treatment of PC12 cells with ATEO increased the expression of anti-oxidant proteins. PC12 cells were treated with ATEO (1.5, 5 and 15 *μ*g/mL) or ZLN005 (2 *μ*M) for 48 h. Total RNAs were isolated from cultured PC12 cells and then reversed transcribed into cDNAs for the detection of mRNAs encoding SOD1, SOD2, GPx1, UCP2 and UCP3 by real-time PCR analysis. *β*-actin served as internal control. Values are expressed as fold of control (untreated culture), and mean ± SEM, where *n* = 3. ^*∗*^*p* < 0.05, ^*∗∗*^*p* < 0.01 compared with control group.

Next, we examined the expression of PGC-1*α* and PGC-1*α*-regulated downstream genes that encode several antioxidant proteins in PC12 cells. Application of ATEO (1.5, 5, and 15 *μ*g/mL) in cultures induced mRNA and protein expression of PGC-1*α* in a concentration-dependent manner: the maximal induction of >100% increase was observed at ∼15 *μ*g/mL (Figure 4). The antioxidant proteins induced by PGC-1*α* included SOD1, SOD2, GPx1, UCP2, and UCP3. In the study, the treatment of PC12 cells with ZLN005 (2 *μ*M, a compound that stimulates the expression of PGC-1*α* and downstream genes) or ATEO (15 *μ*g/mL) stimulated the expression of SOD1, SOD2, GPx1, UCP2, and UCP3 by 1 to 2 folds, respectively, when compared with control group (Figure 5).

### 3.5. Upregulation of PGC-1*α* Expression Was Involved in the Cytoprotective Effects of ATEO

**Figure 6:**
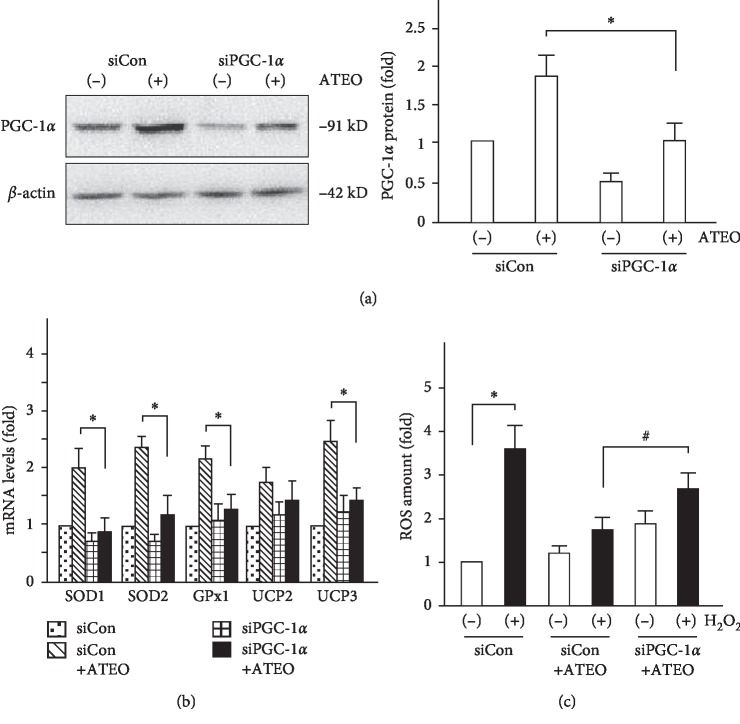
Up-regulation of PGC-1*α* expression was involved in the cytoprotective effects of ATEO in PC12 cells. To elucidate the specificity of ATEO-induced PGC-1*α* expression in cytoprotection, the protein level of PGC-1*α* (a), mRNA level of anti-oxidant proteins (b) and intracellular ROS level (c) were assessed in PGC-1*α* knockdown cells, as earlier mentioned. ATEO (15 *μ*g/mL) was used in the study. *β*-actin served as an internal control. Data are expressed as fold of control (untreated culture), and mean ± SEM, where *n* = 3. ^*∗*^*p* < 0.05, ATEO-treated siCon vs. ATEO-treated siPGC-1*α* group; ^*∗*^*p* < 0.05, siCon vs. H_2_O_2_-treated siCon group; ^#^*p* < 0.05, H_2_O_2_-treated (siCon + ATEO) vs. H_2_O_2_-treated (siPGC-1*α* + ATEO) group.

To elucidate the specificity of ATEO-induced PGC-1*α* expression in cytoprotection, we explored the effect of ATEO on regulation of PGC-1*α*, antioxidant proteins, and intracellular ROS in PC12 cells. PGC-1*α* protein expression was increased by the treatment of ATEO (15 *μ*g/mL), whereas the protein amount was attenuated by PGC-1*α* specific siRNA knockdown (Figure 6(a)). The expression of antioxidant proteins (SOD1, SOD2, GPx1, UCP2, and UCP3), which was upregulated by ATEO treatment, was attenuated by PGC-1*α* specific reduction (Figure 6(b)). In addition, the ROS levels, which were reduced by ATEO pretreatment, were also increased by PGC-1*α* specific reduction in H_2_O_2_-stressed PC12 cells (Figure 6(c)). These results suggested that ATEO attenuated H_2_O_2_-induced cell injury by upregulating PGC-1*α* expression in PC12 cells.

### 3.6. Activation of CREB Was Involved in the Cytoprotective Effects of ATEO

**Figure 7:**
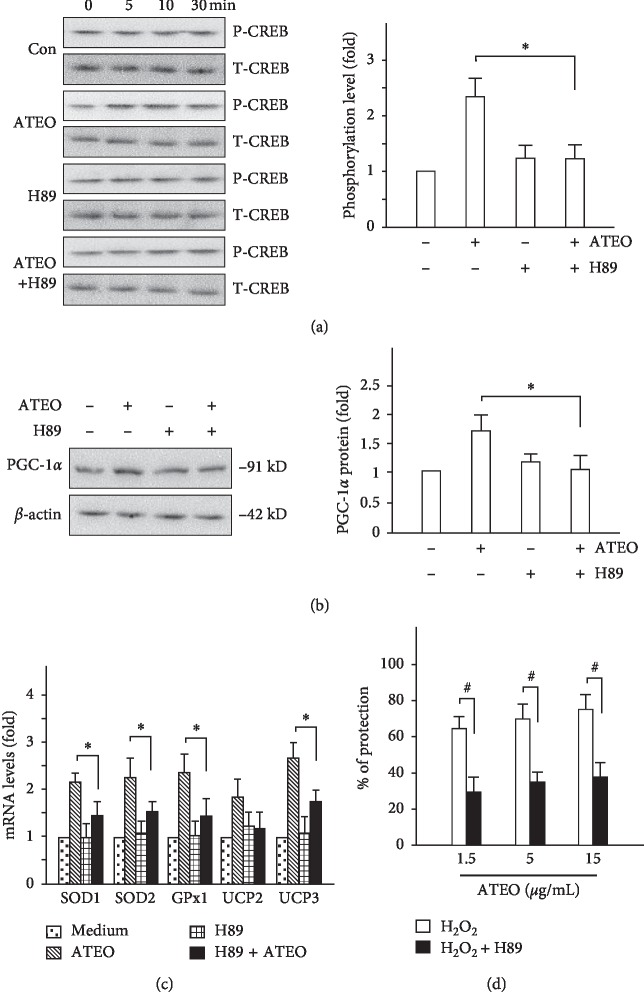
Activation of CREB was involved in the cytoprotective effects of ATEO in PC12 cells. To further elucidate the molecular mechanism underlying ATEO-induced PGC-1*α* expression and cytoprotection is CREB-dependent, the phosphorylation level of CREB (a), protein level of PGC-1*α* (b), mRNA level of anti-oxidant proteins (c) and MTT assay (d) were assessed in H89 (5 *μ*M) pretreated-PC12 cells, as earlier mentioned. ATEO (15 *μ*g/mL) was used in the study. *β*-actin served as an internal control. Data are expressed as mean ± SEM, where *n* = 3. ^*∗*^*p* < 0.05, ^*∗∗*^*p* < 0.01 ATEO-treated vs. (H89 + ATEO)-treated group; ^#^*p* < 0.05, H_2_O_2_-treated ATEO vs. H_2_O_2_-treated (H89 + ATEO) group.

The regulation of PGC-1*α* expression by CREB has been reported [7]. Here, we analyzed whether the molecular mechanism underlying ATEO-induced PGC-1*α* expression and cytoprotection is CREB-dependent. In ATEO (15 *μ*g/mL)-treated PC12 cells, the phosphorylation of CREB at Ser-133 was considerably increased at 5, 10, and 30 min as compared with control group, whereas the phosphorylation levels were attenuated by PKA inhibitor (H89, 5 *μ*M) pretreatment (Figure 7(a)). Consequently, CREB inactivation by treatment with H89 in ATEO-treated PC12 cells led to a decrease in PGC-1*α* protein expression (Figure 7(b)), followed by reduction in the expression of antioxidant proteins (SOD1, SOD2, GPx1, UCP2, and UCP3; see Figure 7(c)). Furthermore, CREB inactivation caused an impairment of cytoprotective effect of ATEO against H_2_O_2_-induced oxidative stress, as assessed by MTT assay (Figure 7(d)). Therefore, ATEO may protect H_2_O_2_-induced cell injury via CREB/PGC-1*α* activation in PC12 cells.

## 4. Discussion

Data from World Alzheimer Report 2018 indicate that while people around the world are living longer, the incidence of neurological disorders, including dementia, Alzheimer disease, Parkinson disease, amyotrophic lateral sclerosis, and stroke, continues to increase and is expected to double every 20 years [31]. An accumulating body of evidence suggests that oxidative stress, an imbalance between cellular production of ROS and the counteracting antioxidant mechanisms, plays a key role in occurrence and progression of neurological disorders [32–34]. Thus, finding ways to produce neuroprotection by modulating oxidative stress is of great importance.

ATR, a traditional Chinese herbal medicine, has been used for years to treat neurological disorders in China. The primary active fraction of ATR is the essential oil. ATEO was reported to exert neuroprotective effects in neurological disorders, including the protection against oxidative stress [23, 24]. However, the cellular mechanism of ATEO against oxidative stress in neuronal cells has not been fully clarified. In this work, we have identified ATEO-mediated cytoprotective mechanism that involves CREB/PGC-1*α* activation in H_2_O_2_-stressed PC12 cells.

PGC-1*α* is an inducible transcription coactivator of redox homeostasis that modulates the expression of many cytoprotective proteins to improve cell survival during stress [35–38]. PGC-1*α* expression is regulated by myocyte enhancer factor 2 (MEF2), forkhead box O1 (FoxO1), activating transcription factor 2 (ATF2), and CREB [39]. Amongst all these transcription factors, CREB is proven to be involved in PGC-1*α*–mediated suppression of ROS and neurodegeneration [7]. Nerve stimulation induces CREB phosphorylation at Ser-133, which consequently recruits CREB binding protein (CBP) and its close paralogue p300 complex to CRE sequence of PGC-1*α* promoter region to promote histone acetylation and activates PGC-1*α* transcription [40]. PGC-1*α* could serve as an adaptive regulator, capable of providing cytotoxic protection from the ensuing consequences of increased ROS activation [41]. In this study, we have confirmed that PGC-1*α* and CREB are part of the same signaling pathway involved in ATEO-mediated cytoprotection by using PGC-1*α* specific siRNA and CREB specific inhibitor. The results regarding CREB/PGC-1*α* activation involved in ATEO-mediated cytoprotection could be concluded here: (i) PGC-1*α* siRNA knockdown led to decreased expression of antioxidant proteins under ATEO treatment, followed by increased level of intracellular ROS after H_2_O_2_ stress; (ii) CREB inactivation by H89 led to decreased expression of PGC-1*α* and antioxidant proteins under ATEO treatment, followed by reduced ATEO-mediated cytoprotective activity after H_2_O_2_ stress.

The regulation of antioxidative proteins studied in this work included SOD1, SOD2, GPx1, UCP2, and UCP3. The upregulation of SOD1, SOD2, and GPx1 could protect cells by scavenging ROS in mitochondria, cytoplasm, and peroxisomes, while the upregulation of UCP2 and UCP3 could further decrease mitochondrial generation of ROS by uncoupling [42]. Our results indicated that ATEO prevented H_2_O_2_-induced cell injury in PC12 cells by upregulating these antioxidative proteins. The neuroprotective effect was also revealed in another report that ATEO prevented oxidative stress-induced cell injury in cultured astrocytes [24]. These results indicate that ATEO may exert antioxidative effects in both neuronal cells and astrocytes, which are the two most important cells in central nervous system. Similar results are also shown in the study of other neuroprotective oils, e.g., lavender oil. It is reported that lavender oil exerts neuroprotective activities on central nervous system and the underlying mechanism may be related to modulation of the NMDA receptor, the serotonin transporter, and neurotoxicity induced by oxidative stress [43–45]. In addition, the ATEO concentration used in the study also showed physiological significance, including promoting the expression and secretion of neurotrophic factors in cultured astrocytes and potentiating nerve growth factor-induced neuronal differentiation in PC12 cells [46, 47]. Therefore, ATEO presents various neurological activities on central nervous system in physiological and pathological conditions. These activities of ATEO may be relevant to the traditional use of ATR: regaining consciousness, tranquilizing the mind, eliminating dampness, and invigorating the circulation of blood [48].

An evaluation of the major chemical constituents present in ATEO that were neuroprotective was also performed. The asarones, the major chemicals present in the oil, were shown to possess antioxidant properties in various animal seizure models [49]. However, asarones were considered as toxic for their genotoxicity and carcinogenicity at high dosage [50]. Thus, the doses of asarones and asarone-enriched extracts should be studied appropriately in order to avoid toxicity. *α*-Asarone and *β*-asarone (1–15 *μ*g/mL) were shown to prevent oxidative stress-induced cell injury in cultured astrocytes [24]. Furthermore, the combination of asarones and eugenols in ATR at 3 : 1–5 : 1 was reported to help enhance therapeutic efficacy and reduce side or adverse effects in dementia mice [51]. These results were consistent with our finding that the combination of asarones and eugenols in ATEO was ∼4.40 : 1 according to our GC/MS data. Thus, it is reasonable to use ATEO for neuroprotection. Furthermore, much more effort is required to elucidate the relationship of composition structure and functional activity of ATEO in redox response.

## 5. Conclusions

In this study, the cytoprotective effects of ATEO against oxidative stress were investigated by using a H_2_O_2_-stressed neuronal cell model. It was shown that ATEO notably induced the expression of antioxidant proteins including SOD1, SOD2, GPx1, UCP2, and UCP3. The underlying mechanism of this action may be related to the upregulatory effect of ATEO on CREB/PGC-1*α* signaling in H_2_O_2_-stressed PC12 cells. These findings provide a molecular insight into the effect of ATEO on cytoprotection, which suggest that ATEO might be developed as a potential neuroprotective agent; further research should focus on validating ATEO as a neuroprotective agent in primary neuron and animal models.

## Figures and Tables

**Table 1:** Chemical composition of ATEO by GC/MS.

Peak no.	*t*_R_/min	Compound	Formula	M_r_	Relative content (%)
1	6.20	2-Hexadecanol	C_16_H_34_O	242	0.06
2	9.84	Camphor	C_10_H_16_O	152	0.34
3	10.38	Borneol	C_10_H_18_O	154	0.34
4	16.08	Ylangene	C_15_H_24_	204	0.06
5	16.91	Longifolene	C_15_H_24_	204	0.16
6	17.81	*α*-Acorenol	C_15_H_26_O	222	0.15
7	19.08	Longifolene-(V4)	C_15_H_24_	204	0.76
8	19.71	Isoledene	C_15_H_24_	204	0.30
9	21.03	Methyl eugenol	C_11_H_14_O_2_	178	**17.10**
10	21.53	2-Allyl-1,4-dimethoxybenzene	C_11_H_14_O_2_	178	0.28
11	22.02	Cedrene	C_15_H_24_	204	0.07
12	22.68	*β*-Guaiene	C_15_H_24_	204	0.10
13	22.97	Patchoulene	C_15_H_24_	204	0.57
14	23.36	*α*-Muurolene	C_15_H_24_	204	0.57
15	24.26	Shyobunone	C_15_H_24_O	220	0.36
16	24.86	*β*-Cadinene	C_15_H_24_	204	0.07
17	26.03	*α*-Calacorene	C_15_H_20_	200	0.04
18	27.19	Elemicin	C_12_H_16_O_3_	208	0.15
19	28.38	*γ*-Asarone	C_12_H_16_O_3_	208	**2.39**
20	32.04	*β*-Asarone	C_12_H_16_O_3_	208	**68.25**
21	32.91	8-Acetyl-5,5-dimethyl-nona-2,3,8-trienoic acid, methyl ester	C_14_H_20_O_3_	236	0.02
22	33.86	*α*-Cadinol	C_15_H_26_O	222	0.17
23	34.09	1,2-Dimethoxy-4-(2-methoxy-1-propenyl)benzene	C_12_H_16_O_3_	208	0.03
24	35.39	Spiro[4.5]dec-6-en-8-one, 1,7-dimethyl-4-(1-methylethyl)-	C_15_H_24_O	220	0.06
25	36.42	*α*-Asarone	C_12_H_16_O_3_	208	**4.65**

## Data Availability

The data used to support the findings of this study are included within the article.

## Supplementary Materials

Supplementary Materials Supplementary Figure 1. Effect of ATEO on the growth of PC12 cells. Cultured PC12 cells were treated with ATEO (0-50 *μ*g/mL) for 48 h. A cell viability and proliferation test (using the colorimetric MTT assay) was performed. Data are expressed as mean ± SEM, where n = 5. ^*∗*^*p* < 0.05 compared with control group. Supplementary Figure 2. Various pretreatment times of ATEO before H2O2 administration. Cultured PC12 cells were pretreated with ATEO (15 *μ*g/mL) for various hours before H2O2 (400 *μ*M) addition for 24 h. A cell viability and proliferation test (using the colorimetric MTT assay) was performed. Data are expressed as mean ± SEM, where n = 5. ^#^*p* < 0.05 compared with the H2O2-treated group.
