# Supplementary material for: The Essential Oil from Acori Tatarinowii Rhizome (the Dried Rhizome of Acorus tatarinowii Schott) Prevents Hydrogen Peroxide-Induced Cell Injury in PC12 Cells: A Signaling Triggered by CREB/PGC-1α Activation
Source: Evid Based Complement Alternat Med. 2020 Mar 9;2020:4845028. doi: 10.1155/2020/4845028 (PMC7085381; doi:10.1155/2020/4845028)
Supplement: Supplementary Materials — Supplementary Figure 1. Effect of ATEO on the growth of PC12 cells. Cultured PC12 cells were treated with ATEO (0-50 μg/mL) for 48 h. A cell viability and proliferation test (using the colorimetric MTT assay) was performed. Data are expressed as mean ± SEM, where n = 5. ∗p < 0.05 compared with control group. Supplementary Figure 2. Various pretreatment times of ATEO before H2O2 administration. Cultured PC12 cells were pretreated with ATEO (15 μg/mL) for various hours before H2O2 (400 μM) addition for 24 h. A cell viability and proliferation test (using the colorimetric MTT assay) was performed. Data are expressed as mean ± SEM, where n = 5. #p < 0.05 compared with the H2O2-treated group. [file 4845028.f1.docx]

**
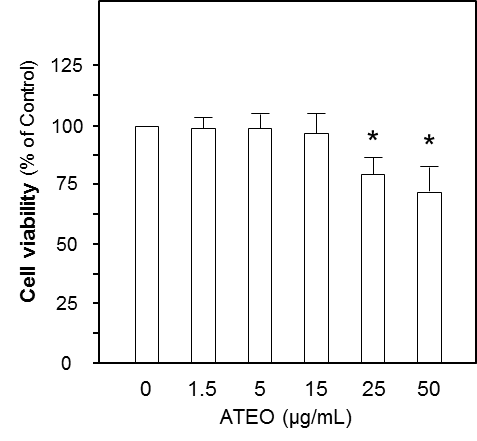
**

SUPPLEMENTARY FIGURE 1: Effect of ATEO on the growth of PC12 cells. Cultured PC12 cells were treated with ATEO (0-50 μg/mL) for 48 h. A cell viability and proliferation test (using the colorimetric MTT assay) was performed. Data are expressed as mean ± SEM, where *n* = 5. **p* < 0.05 compared with control group.


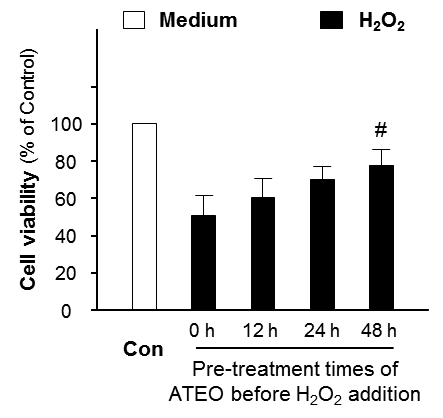


SUPPLEMENTARY FIGURE 2: Various pre-treatment times of ATEO before H_2_O_2_ administration. Cultured PC12 cells were pre-treated with ATEO (15 μg/mL) for various hours before H_2_O_2_ (400 μM) addition for 24 h. A cell viability and proliferation test (using the colorimetric MTT assay) was performed. Data are expressed as mean ± SEM, where *n* = 5. ^#^*p* < 0.05 compared with H_2_O_2_-treated group.
